# Photon management for augmented photosynthesis

**DOI:** 10.1038/ncomms12699

**Published:** 2016-09-01

**Authors:** Matthew D. Ooms, Cao Thang Dinh, Edward H. Sargent, David Sinton

**Affiliations:** 1 Department of Mechanical and Industrial Engineering and Institute for Sustainable Energy, University of Toronto, 5 Kings College Rd., Toronto, Ontario, Canada M5S3G8; 2 Department of Electrical and Computer Engineering, University of Toronto, 10 King's College Rd., Toronto, Ontario, Canada M5S3G4

## Abstract

Microalgae and cyanobacteria are some of nature's finest examples of solar energy conversion systems, effortlessly transforming inorganic carbon into complex molecules through photosynthesis. The efficiency of energy-dense hydrocarbon production by photosynthetic organisms is determined in part by the light collected by the microorganisms. Therefore, optical engineering has the potential to increase the productivity of algae cultivation systems used for industrial-scale biofuel synthesis. Herein, we explore and report emerging and promising material science and engineering innovations for augmenting microalgal photosynthesis.

## Abstract (web summary)

Photosynthetic microalgae could provide an ecologically sustainable route to produce solar biofuels and high-value chemicals. Here, the authors review various optical management strategies used to manipulate the incident light in order to increase the efficiency of microalgae biofuel production.

## Graphical abstract

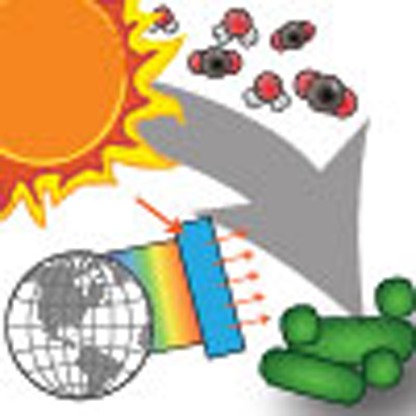

Photosynthesis is the model process for storing solar energy in complex chemical bonds. Annually it results in the fixation of upwards of 120 billion tons of carbon through terrestrial plants alone^1^, and nearly as much again inside the world's oceans^2^. Humankind has sought over decades to mimic this process synthetically; however, to date, photosynthesis remains the only option for the sustainable production of many complex chemicals. In particular photosynthesis provides a sustainable path for the synthesis of high-energy-density liquid biofuels—an important priority in a world increasingly stressed by anthropogenic CO_2_. For these reasons, cultivation of photosynthetic plants and microalgae for biofuel production has attracted great interest.

Biofuel production from microalgae can follow several routes. Biodiesel can be produced by reacting triacylglycerols (a type of cellular energy storage lipid) with an alcohol, such as methanol, to produce fatty acid methyl esters (biodiesel), a process known as transesterification. For biodiesel production green algae and diatoms show particular promise as feedstocks owing to their high lipid concentration which can exceed 50% of the cell's dry-weight^3^. In addition, microalgal carbohydrates can be converted to biomethane or biohydrogen, through anaerobic digestion or to bioethanol through fermentation^4^. Alternatively, raw biomass in its entirety can be converted into biocrude oil using thermochemical conversion processes such as pyrolysis or hydrothermal liquifaction^4^. To avoid harvesting and processing of raw biomass for biofuel extraction, direct photobiological production of hydrogen is possible with certain species, such as *Chlamydomonas reinhardtii*^5^. With genetic modifications, particularly of cyanobacteria, other biofuels and biofuel precursors can similarly be evolved including isobutyraldehyde^6^, isobutanol^6^, 1-butanol^7^ and isoprene^8^. Bio-electricity production in microalgal bio-photovoltaic cells has also been demonstrated^9^,^10^. This suite of microalgal energy generation options is a distinct advantage of photosynthesis over other solar energy conversion techniques which are typically constrained to generate only simple products, as in the case of photovoltaic electrolysis. The simultaneous production of a variety of co-products in addition to biofuel adds further value and flexibility to microalgal cultivation (Fig. 1; Box 1; Table 1). The generation of sufficient quantities of biomass is a prerequisite towards meeting large-scale demand and achieving economic viability, and is predicated on efficient sunlight utilization during cultivation. Present-day operations typically exhibit photosynthetic energy conversion efficiencies of about 1%, far short of the theoretical maximum of approximately 12% (ref. 11), owing to energy losses at all stages of the process (Fig. 1). As a result, industrial-scale cultivation of photosynthetic microorganisms has yet to achieve economic viability. Improving this efficiency is a multivariable problem, aspects of which have been previously discussed in several excellent reviews addressing microalgal culturing techniques^12^, economics^13^ and applications^14^.

In this review, we explore the latest optical engineering advances to manage light during microalgae cultivation to increase conversion efficiency and maximize productivity, summarized in Table 2. Latitude and weather determine the solar resources available and consequently the maximum productivity. The spectral distribution and intensity of light can be tuned using modern materials, reactor configurations and light sources, while mixing, culture density and light path lengths can be adjusted to optimize the light intensity experienced by the suspended cells, both spatially and temporally. In addition, the physical size and composition of the cellular light-harvesting apparatus itself can be tailored to enhance the spectral sensitivity and tolerance of cells to varying light environments. Material science, engineering and bioengineering approaches can provide elegant solutions to increase the efficiency of bioproduct production by photosynthetic organisms and potentially bypass the hurdles facing fully artificial photocatalytic devices.

## Managing light collection

### Reactor location selection

**Figure 1:**
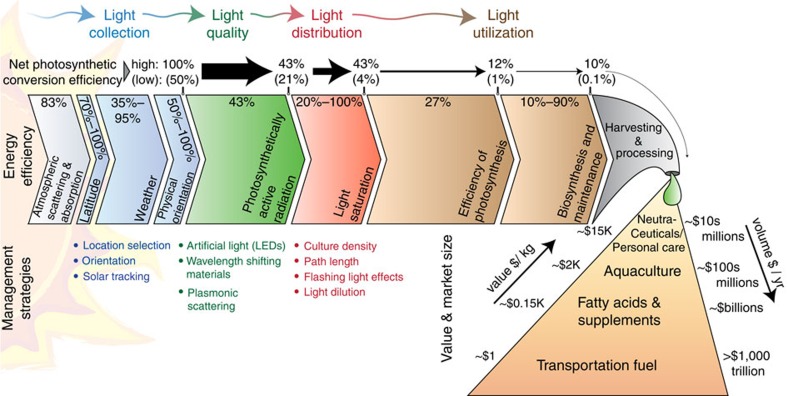
Sunlight-to-biomass conversion efficiency and strategies. The amount of useful energy decreases between the sun and the final bio-product. Initial atmospheric scattering leads to attenuation of direct beam and diffuse light of around 17%, an effect which increases with latitude by as much as an additional 30%. Weather conditions can result in an additional 65% loss based on the difference between clear sky and measured irradiances of several representative cities around the world (Fig. 2a). The physical orientation of the culture unit relative to the sun can reduce the irradiation intensity by another 50% for a horizontal surface compared with a surface oriented directly towards the sun. Upon reaching the culture, 57% of typical sunlight is not useful for photosynthesis and is therefore considered to be lost. At peak sun intensity, as much as 80% more of the absorbed sunlight may be wasted since it exceeds the saturation limit of the photosynthetic microorganisms. Additional losses related to energy transduction through the photosynthetic apparatus (73%) and biomass synthesis and maintenance (10%–90%) results in further losses^11^. Net photosynthetic efficiency, calculated from when light is incident on a reactor surface to its storage as a simple carbohydrate, can range between 0.1% and 10%. Of the markets that microalgal products can serve, transportation fuel represents the largest by volume and has few sustainable alternatives. However, from a value perspective transportation fuel is relatively inexpensive making alternatives difficult to justify economically. Higher value products such as dietary supplements, aquacultures feedstocks, additives for personal care products, and nutraceuticals have substantially lower demand, but can be orders of magnitude more valuable. Technology maturation can be supported by including high-value products production to support low-value, high-volume production of energy. Approximate values based on refs 14, 98.

**Figure 2:**
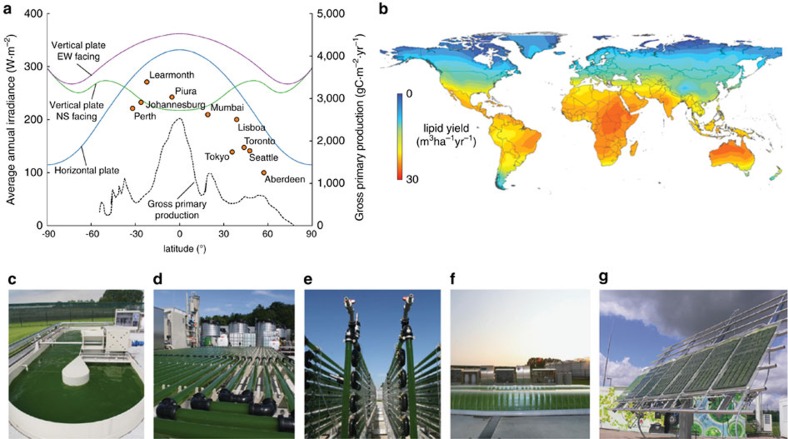
Managing solar light collection for microalgal cultivation. (**a**) Average annual solar irradiance by latitude based on clear sky models (solid lines) for different flat plate photobioreactor orientations. Also shown are actual measured irradiances (circles) at different locations around the globe showing the impact of weather and atmospheric conditions. The global gross primary production in grams of carbon sequestered per square metre per year (g_C_·m^−2^·yr^−1^) from photosynthesis by latitude (black dashed line) shows strong correlation to irradiance. Gross annual production is the average result from several different data-driven models using the model tree ensemble, artificial neural network, light use efficiency, and the Köppen-Geiger cross Biome approaches. (**b**) Estimation of global annual lipid productivity based on growth models typical of *Nannochloropsis*. Results are interpolated using data from 4,388 locations around the globe and account for the availability of resources, including light, for microalgal cultivation. (**c**–**f**) Examples of different photobioreactor configurations at the Algae Production and Research Center (AlgaePARC) in the Netherlands showing examples of pilot scale (**c**) raceway (volume=4,730 L), (**d**) horizontal tubular (volume=560 L), (**e**) vertical tubular (volume=1,060 L) and (**f**) flat plate (volume=390 L) photobioreactors. (**g**) A solar-tracked photobioreactor in Hamburg-Reitbrook, Germany (volume=263 L). (**a**) gross primary production data adapted from ref. 1, irradiance data retrieved from Meteonorm Global Meteorological Database (http://meteonorm.com/), (**b**) adapted with permission from ref. 21. (**c**–**f**) Reproduced from ref. 22 with permission from Elsevier (**g**) reproduced from ref. 24 with permission from Springer.

Solar resources vary by geographic region and are a determinant of a location's suitability for microalgal culture. The simplest assessments of solar resources use clear sky irradiance models^15^, which primarily account for variation in solar flux due to atmospheric scattering which can reduce sunlight by a factor of around 83% at the equator (Fig. 1). Scattering losses, measured in terms of direct beam plus diffuse radiation, increase with distance from the equator incurring an additional efficiency factor of up to 70% at the poles. These models are limited in their predictive power however, because they overlook the impact of weather which can attenuate the available solar energy by a further factor of 35% (Fig. 1), based on measured monthly average irradiances (direct beam and diffuse) at several locations around the world, shown in Fig. 2a.

Site selection studies are therefore making increased use of historic meteorological data to project future solar resources in a region^13^,^16^. An assessment of Western Australia included average daily solar fluxes ranging between 150 and 277 W m^−2^, with over 90% of the continent between 208 and 277 W m^−2^ (ref. 17). This regional variation in sunlight can drive an increase in productivity from 3.6 to 7.7 g m^−2^ per day based on historical productivity observed in *Chrysotila criteria* and *Tetraselmis spp*^17^. Microalgae farming assessments in Chile identified primarily coastal desert regions as suitable locations for large-scale culture facilities using 165 W m^−2^ average annual irradiance as the threshold minimum^18^. Increasing access to historical irradiance data for thousands of locations worldwide has enabled greater insight into global weather trends and irradiances driving improvement in global site selection assesments^19^,^20^. Figure 2b shows a map of lipid productivity potential based on global irradiance data. Owing to high annual irradiances, minimal cloud cover and warm temperatures, equatorial locations have consistently shown the highest potential for microalgae cultivation^21^, correlating with the global trend in primary photosynthetic production (Fig. 2a)^1^. Based on growth models developed for *Nannochloropsis sp*. the global biomass production potential was estimated to be 9.4 g m^−2^ per day, with the maximum occurring in Australia (18 g m^−2^ per day) (ref. 21). The significant variation in solar resources by location makes these detailed site selections studies, which also account for other required resources (for example: water, carbon, labour and access to infrastructure), an important instrument in the planning and implementation of future microalgae cultivation projects.

### Reactor orientation and solar tracking

Outdoor photobioreactors with typical cell density loading typically have light path lengths on the order of centimeters for enclosed photobioreactors, or tens of centimetres for open ponds (Fig. 2c–g)^22^. For flat plate designs (with large illuminated surface areas relative to their thickness) the direction in which they face is a significant factor in the amount of incident light they intercept. Models and tools for calculating the irradiance incident on a plane surface are now common in the solar energy field and can be used to calculate the irradiance potential for a given location and orientation^23^. An important difference is however, that unlike typical photovoltaic panels, photobioreactors are most often able to utilize light incident on both their front and back surfaces, allowing them to capture both direct and diffuse light.

Continuous tracking of the sun ensures maximal collection of sunlight. Based on clear sky models, the average annual intensity on a horizontal surface compared with a solar-tracked surface (example shown in Fig. 2g) is reduced by about 50% for latitudes of ±60° and about 77% at the equator (Fig. 1). Real-world examples of plate photobioreactors in France (47° N lat.) and Sudan (19 °N lat.) showed that horizontal reactors experienced 72 and 73% of the annual average irradiance seen by solar-tracked panel reactors, respectively^20^. In Germany (53 °N lat.), horizontal photobioreactors saw only 69% of the average annual irradiance seen by a solar-tracked photobioreactor^24^.

For stationary photobioreactors a vertical orientation facing east–west will generally intercept more light on average over the course of a year compared with both horizontal and north–south facing surfaces, as seen from clear sky models (Fig. 2a) and corroborating field tests which showed over 60% increased light harvesting during the summer for a location in Spain (37° N Lat.)^25^. Periodic adjustments in the orientation of flat plate reactors during the year can maximize the collected sunlight without the additional cost of continuous tracking^26^. For a site in Israel (31° N lat.), adjusting the tilt angle of a south facing flat plate reactor four times each year resulted in a 35% increase in productivity compared with a stationary horizontal reactor^26^.

The benefits of orienting a photobioreactor towards the sun to increase the amount of intercepted light are most simply realized for small-scale installations. For larger installations that involve arrays of reactors, the effect of shading between reactors becomes significant. While vertically oriented reactors intercept more light in isolation, they also create shaded areas limiting the light available to adjacent reactors. This shading can affect the productivity of large-scale installations and makes the spacing and height of individual reactors an important design criteria^27^,^28^. Furthermore, for these large installations the greater need for inter-reactor spacing results in additional loss when light falls upon the non-photosynthetic surfaces between reactors.

For large-scale systems, maximum light collection is most simply achieved through horizontal orientation of large area reactors or ponds, whereas for smaller single reactors or one-dimensional arrays where shading is of little concern, vertical orientation is preferred. Harvesting as much solar energy as possible is crucial for maximizing overall areal productivity, but high light intensity can also result in photoinhibition and energy loss through non-photochemical quenching pathways, reducing overall efficiency. Combining effective light collection with light distribution strategies, which will be discussed later in this review, is important to make full use of the collected photons.

## Managing spectral distribution

### Effect of spectral distribution

Once photons are intercepted by the cultivation apparatus, there is an opportunity to influence the spectral distribution of the transmitted photons. Photoautotrophs have evolved an array of light-harvesting pigments that can absorb energy across the visible spectrum, and the composition of these pigments determines the wavelengths that are absorbed for photosynthesis. Photosynthetically active radiation (PAR) is conventionally limited to wavelengths between 400 and 700 nm. This portion of the spectrum accounts for approximately 43% of the solar energy incident on earth and 28% of solar photons. Even though it is common to consider all PAR photons to be equally adept at driving photosynthesis, the array of chromophores involved in light harvesting and phytochromes involved in light sensing suggest a more complicated picture.

**Figure 3:**
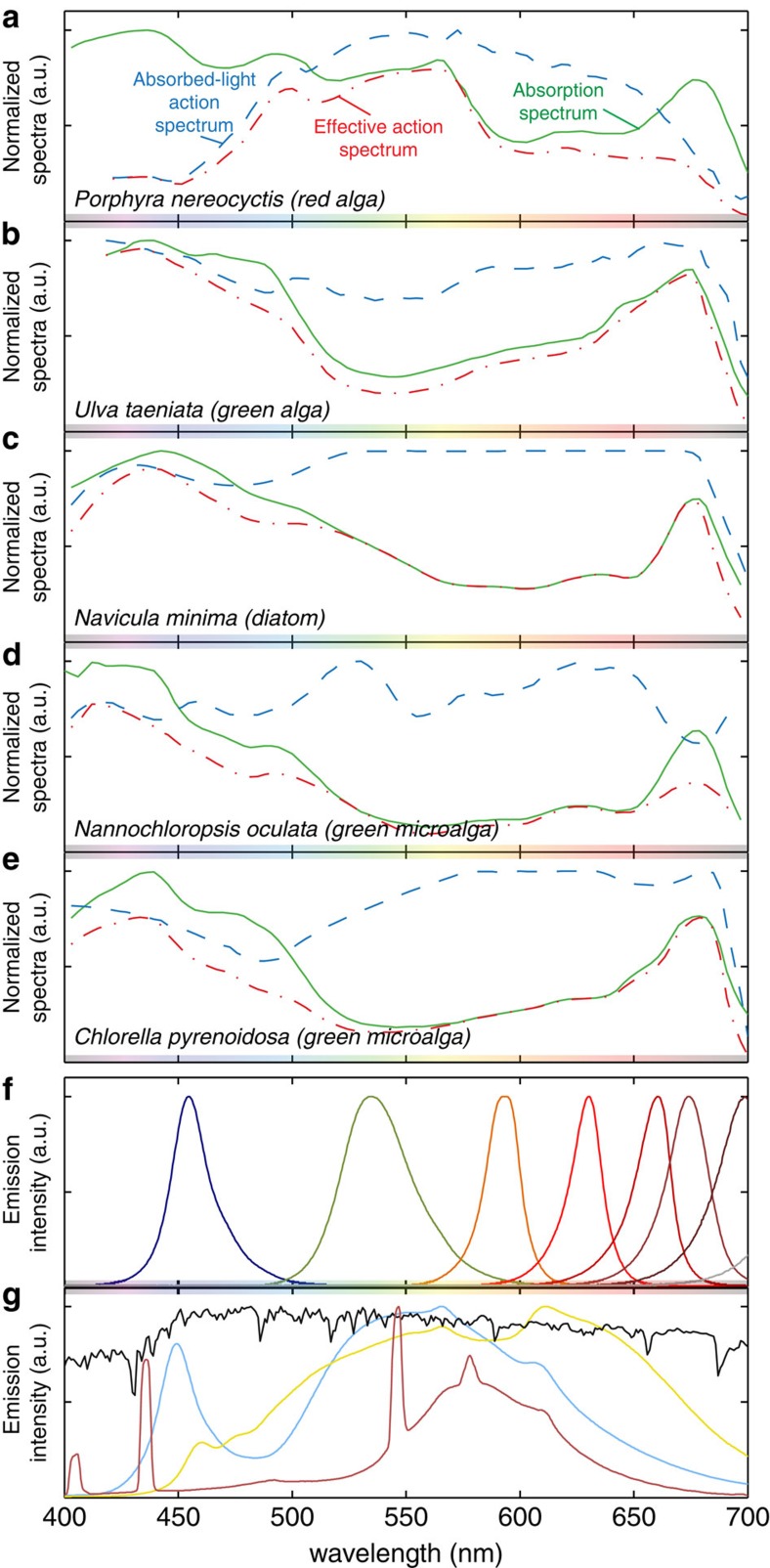
Light absorption and emission. (**a**–**e**) Normalized absorption spectra (green solid lines), absorbed-light action spectra based on measured oxygen evolution (blue dashed lines), and the combined effective action spectra (red dash-dot line) determined as the product of the absorption and absorbed-light action spectra for macroalgae and microalgae. (**f**) Emission spectrum of typical monochromatic and (**g**) white light emitters with sunlight (black solid line) included as a reference. While different qualities of white light can be produced using, for example, cool white LEDs (blue solid line), warm white LEDs (yellow solid line) or fluorescent bulbs (red solid line), the spectra emitted from these light sources are drastically different from the light emitted by the sun, and will consequently induce different responses from photosynthetic organisms. Action and absorption adapted from (**a**,**b**) ref. 29, Copyright 1950 Rockefeller University Press, (**c**) ref. 99, (**d**) ref. 31 with permission from Elsevier, (**e**) ref. 100 and (**f**,**g**) emission spectra as measured by the authors, solar spectrum from AM1.5.

The action spectrum of photosynthetic organisms describes the rate of photosynthesis in response to light at different wavelengths^29^, and is typically determined by measuring autofluorescence, oxygen generation or growth rate under low-intensity monochromatic light. Figure 3a–e shows examples of both the absorption and action spectra for several macroalgae and microalgae species. In particular, Fig. 3 highlights the difference between a species' ability to absorb specific wavelengths versus how well that species can utilize this absorbed light. Only a limited number of studies have measured the action spectrum for a small number of microalgae, while most others use the absorption or attenuation (absorption and scattering) spectrum as a proxy. Figure 3 shows however that there is often significant variation between the absorption and the absorbed action spectra for many classes of micro and macroalgae, and it is therefore important to note that wavelengths which may appear to be of low value based on poor absorption, may nevertheless play a substantial role in driving photosynthesis in optically thick cultures where all photons are ultimately absorbed^30^. For example, although the microalgae *Nannochloropsis oculata* (Fig. 3d) has a low absorption rate in the green region, it shows a noticeable peak in its absorbed action spectrum at these wavelengths^31^. In cultures of *Scenedesmus bijuga* and *C. reinhardtii*, green and yellow light respectively outperformed other wavelengths likely due to the combination of a deeper penetration depth for these poorly absorbed wavelengths and their ability to efficiently drive photosynthesis when they are finally absorbed^30^,^32^. For optically thin cultures however, the absorption spectra dictates the overall photon absorption of the culture at each wavelength and transmission of poorly absorbed light leads to lower performance at these wavelengths. For *N. oculata*, because blue light is strongly absorbed it resulted in the highest photosynthesis rate in optically thin cultures at low irradiances (60 μmol_ph_ m^−2^ s^−1^) (ref. 31). For practical applications however, cultures are usually fully absorbing and wavelengths between 500 and 650 nm are most efficient, particularly for cyanobacteria where shorter wavelengths often trigger photo-protection mechanisms.

**Figure 4:**
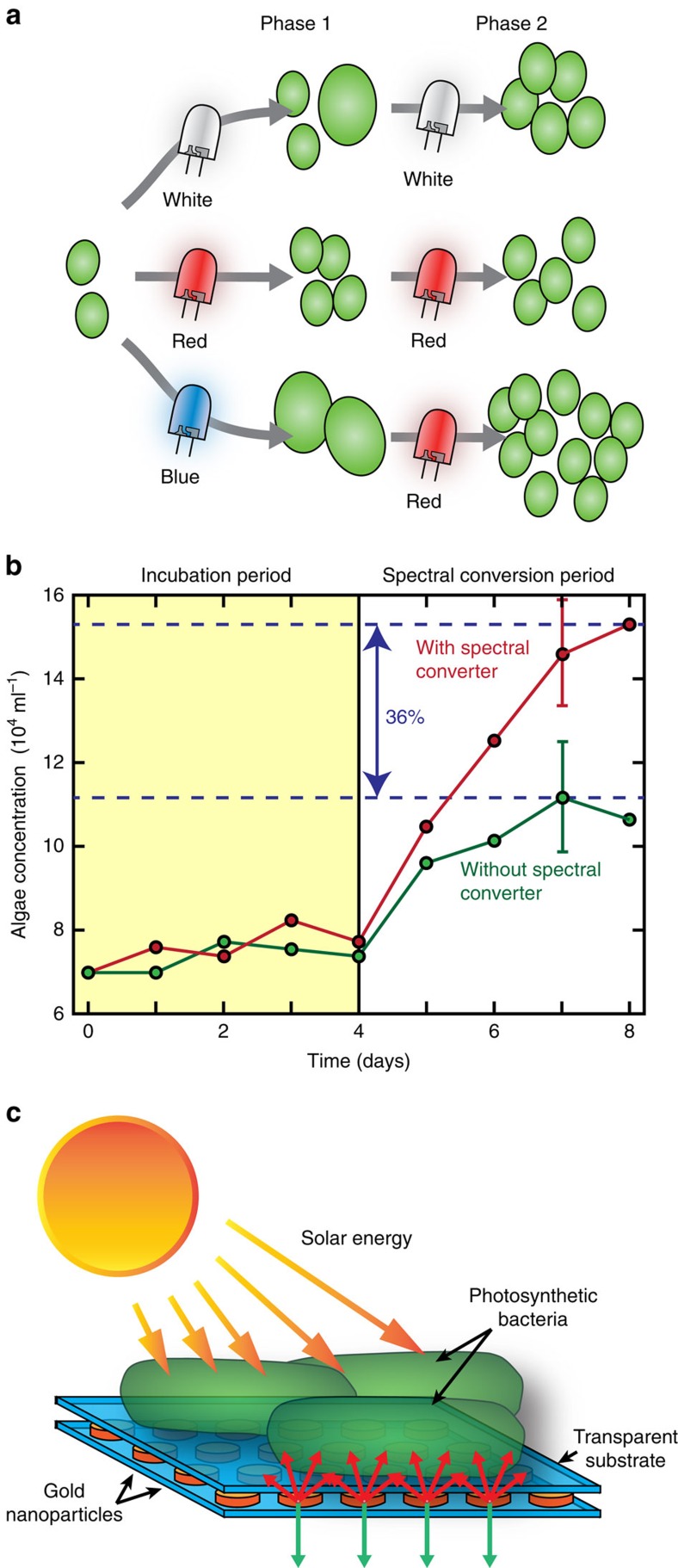
Managing the light spectrum. (**a**) Schematic showing how alternating blue and red light can induce increased growth in green algae by first increasing cell size under blue light and subsequently inducing higher division rate by exposure to red light. (**b**) Graph showing the impact on growth of green algae of using spectral shifting dyes to convert green light to red light in a back-reflecting flat plate photobioreactor. The shaded region represents the initial incubation period after which the spectral converting layer was introduced. A net increase in biomass production of 36% was observed when the converter was used. Error bars represent a counting error of 25,000 cells. (**c**) Schematic showing how plasmonic backscattering can be used to direct photosynthetically useful light (red arrows) towards microalgae cultures while allowing other wavelengths (green arrows) to be transmitted for other applications. This approach uses the wavelength specific nature of plasmonic resonances to selectively scatter light. The resonant wavelength can be tuned by controlling the geometry, distribution, and material of the plasmonic nanoparticles, substrate, and surrounding media. (**a**) Adapted from ref. 38 with permission from Elsevier (**b**) adapted from ref. 45, previously published under a CC-BY-NC-SA license, copyright Wondraczek *et al*., licensee Macmillan Publishers Ltd 2013 (**c**) reproduced from ref. 56 with permission from AIP Publishing.

Many photosynthetic organisms respond to changes in spectral distribution through sophisticated sensory mechanisms to preferentially express desirable metabolites^33^,^34^. Increased amounts of blue light have been shown to increase the expression of chlorophyll in *Chlorella sp*^35^ and lipids in both *Tetraselmis sp*. and *Nannochloropsis sp*^36^. Dynamically adjusting the wavelength distribution during cultivation can further enhance productivity. For *Haematococcus pluvialis*, red light promoted growth while blue light increased astaxanthin expression in the cells^37^. By culturing first with red light and then switching to blue, high biomass productivity and high astaxanthin concentrations were achieved. Similarly, a 20% enhancement in growth of *Chlorella vulgaris* was observed when grown with blue light for two days which resulted in an increase in average cell size, followed by red light for three days which increased the rate of cell division (Fig. 4a) resulting in an overall increase in productivity compared with control cultures^38^. These wavelength induced responses invite new avenues for tailored cultivation protocols using dynamic lighting to maximize production or expression of useful products.

In summary, the photosynthetic activity at any given wavelength is a complex coupling of the efficiency of transfer of the absorbed light energy by the light-harvesting pigments to the photosynthetic reaction centres (action spectrum), the fraction of incident light harvested by the cell (absorption spectrum), and the local light intensity which is affected also by culture density and depth. These relationships are further complicated by adaptation and photoinhibition responses that introduce second order dependencies on light intensity, spectral distribution and the physical environment. To leverage wavelength as a tool to maximize productivity in microalage, additional research needs to be conducted in a systematic fashion to elucidate these relationships more clearly, and define the action spectrum for a broader range of species and culture conditions.

### Light-emitting diodes

High efficiency LED emitters (25%–66% W·W^−1^) (ref. 39) are now readily available and emit at wavelengths spanning the visible spectrum (Fig. 3f). Although other types of artificial illumination have been used for horticultural applications, including high-intensity discharge and fluorescent lamps, LEDs are expected to become dominant due to their long lifetimes, stability, low operating temperature, size and efficiency^40^. Benefits of LED light also include customization of the emitted spectrum and high degrees of spatial and temporal control which can compensate for the intensity, intermittency and day/night cycle of sunlight. Artificial light sources also allow culture vessels to be decoupled from the sunlight collection apparatus, simplifying temperature control, reactor geometry and making reactor positioning more manageable. Control of temperature is particularly important since the reaction rate and regulation of genes involved in the photosynthetic dark-reactions are strongly temperature dependant^41^. In temperate climates outdoor temperatures are often too low to support growth, and the costs of active temperature control for outdoor facilities are energetically and economically prohibitive. In warmer climates, operations may require cooling, which can introduce further challenges.

Energetically, LED-lit microalgal cultures can approach the net photosynthetic efficiencies achieved in outdoor pond reactors. Collecting solar energy with photovoltaic cells (18% efficient) connected to power high efficiency LEDs (approximately 46% efficient), net photosynthetic conversion efficiencies of about 1% could be achieved^42^, which accounts also for the efficiencies of photosynthesis and biosynthesis/maintenance (considered to be 27% and 50%, respectively—see Fig. 1). Photovoltaic electricity generation allows for a direct sunlight-to-biomass efficiency comparison, though on-sight solar farms are not likely feasible. For practical applications, it is more likely however that electricity to operate the LED's would be purchased. Based on a typical cost of electricity of $0.10 per kW·h, an electricity-to-light conversion efficiency of 40%, a light-to-biomass conversion efficiency of 13.5% (27% for photosynthesis and 50% for biosynthesis and maintenance), and a biomass energy content of 23 kJ per g_dry weight_ (ref. 43) the electricity cost of LED grown microalgae is estimated around $14 per kg_dry weight_, which is in agreement with previous studies^42^. This represents a cost of approximately $3,800 per barrel of oil equivalent, two orders of magnitude greater than the price of oil. Clearly, this cost is unacceptable for the production of low-value biofuels. For high-value bioproducts however, and applications with additional value streams such as flue gas or wastewater remediation, the economics improve. For example, astaxanthin can be harvested from *H. pluvialis* in concentrations of 2.5% dry weight or more, and sold for $2,000–$7,000 per kg resulting in a biomass value of $50–$175 per kg_dry weight_. Pond Biofuels (Toronto, Ontario) currently uses LED-lit microalgal cultures to capture CO_2_ from cement plant emissions. In such instances, the additional benefits and convenience of artificial illumination may justify the added expense while still remaining profitable^42^,^44^.

Though commercial applications for LED-lit photobioreactors are limited, LEDs have become ubiquitous in lab-scale microalgal research. Care needs to be taken however when, extrapolating results to outdoor reactors as artificial light sources have spectral distributions and irradiance profiles that differ from solar irradiance (Fig. 3f,g). In addition, measured light intensity emitted from artificial sources can vary widely owing to the distance dependence of LED lights which are typically point sources, intensity scaling inversely with distance cubed. Using arrays of LEDs, collimating optics, or diffusers can help homogenize the light field, but the detailed optics of these configurations are not often reported^33^. Control experiments typically involve white LEDs or fluorescent lamps which can have a range of emission spectra, most of which are poor approximations of sunlight^33^, as shown in Fig. 3g. Additional rigour in addressing and reporting the optical controls and parameters used in microalgae studies should be asserted more consistently in future work.

### Wavelength shifting materials

Converting photons from one wavelength to another using fluorescent or phosphorescent materials can produce a spectrum amenable to increased growth or metabolite expression^45^. Organic and inorganic dyes, phosphors and quantum dots are promising candidates for converting light with little or no photosynthetic potential into light with higher photosynthetic potential^46^. Ideally, materials should be both highly absorbing to harvest a meaningful amount of light, exhibit high conversion efficiencies and have emission spectra sufficiently separate from their absorption spectra so as to avoid reabsorption of converted light.

Down-conversion of light incurs an energy penalty. Nevertheless, the opportunity to harvest photons that would otherwise not be available for photosynthesis is compelling. Most notably, ultraviolet light which is generally detrimental to growth can be converted to visible light usable for photosynthesis. For low-density cultures, converting poorly absorbed light to highly absorbed light (such as green to red) also improves light capture efficiency. For high-density cultures, converting blue light to green can provide greater light penetration into the culture by increasing the fraction of highly penetrating wavelengths, and mitigating the effects of light saturation under high-intensity illumination.

Wavelength shifting of incident light has typically been tested using discrete layers of fluorescent or phosphorescent materials. These converting layers are positioned either between the culture and light source (front-side conversion)^47^,^48^, or behind the culture to capture and convert transmitted light^45^. The latter configuration can be augmented with a reflective backing to reflect all transmitted and converted light back towards the culture, though, is useful only for dilute cultures where a meaningful amount of light can reach the dye layer. An example of this approach is highlighted in Fig. 4b, where a photoluminescent phosphor coated mirror back-plate was used to culture *H. pluvialis* and resulted in 36% more biomass generation at low densities.

For larger scale, outdoor installations, front-side conversion is a more practical approach since typical culture densities do not allow significant light transmission. To date, limited success has been seen growing and expressing pigments in *C. vulgaris* and cyanobacteria *Gloeothece membranacea* using luminescent acrylic sheets to increase the ratio of various portions of the spectrum^49^. Ultraviolet converting dyes entrained in the photobioreactor material can be used harvest and convert ultraviolet light that would otherwise be absorbed by the reactor (or transmitted to the culture) into visible light^50^,^51^. Examples of this approach have shown a 74% increase in biomass productivity when polycarbonate front-side ultraviolet converters were used and illuminated with ultraviolet light, and a 45% increase when acrylic converters were used^50^.

Of particular importance is the efficiency of the spectral tuning layer with respect to light directed towards the cell culture. Many wavelength shifting materials report high internal quantum efficiencies; however, these efficiencies are determined using monochromatic excitation and low concentrations of dye meaning only a small amount of incident light in actually converted. External quantum efficiencies can fall far short of these values as the emitter concentration increases to meaningful densities, due to reabsorption and scattering losses. Furthermore, unless sub-saturating light is used, it is difficult to disambiguate the impact of light attenuation (which alone will have a positive effect on photosynthetic efficiency), and the additional benefit of spectral tuning. Continued work in this field should assess these factors more rigorously, and focus on developing/utilizing the highest performing materials, particularly those that can convert non-PAR photons into PAR photons, with minimal attenuation of visible light.

### Plasmonic scattering

When photons interact with the conduction electrons in metals or metallic nanoparticles, collective oscillations of the electrons can result, called surface plasmons. Exciting surface plasmons in metals significantly enhances the electromagnetic field in the vicinity of the metal surface or particle leading to enhanced absorption or scattering at specific resonant wavelengths. The precise nature and magnitude of these effects is a function of the material type, size, shape and the surrounding media. These optical effects have previously been used to enhance photo-conversion in other fields, particularly photovoltaics, by encouraging useful wavelengths to be redirected into, or confined within, the active media—whether biological or synthetic.

Plasmon enhanced fluorescence has been the subject of intensive research, and recently has been applied to the excitation of the photosynthetic apparatus in whole cells^52^. High-density cyanobacteria biofilms were cultivated in the presence of plasmonically enhanced electromagnetic fields on thin metallic films^52^ in which both light and the cells were confined to the substrate surface. Plasmonic scattering of light can also assist in containing specific wavelengths within photobioreactors^53^,^54^,^55^,^56^. *C. reinhardtii* and *Cyanothece* 51142 showed 30% increased biomass production when plasmonic layers made of silver nanoparticle suspensions scattered blue light back towards the cells^54^. Combinations of nanoparticles with different geometries and materials can produce complex scattering spectra to induced expression of desired metabolites^55^. Nanoparticles adhered to a reactor surface instead of suspended in a solvent can provide similar spectral effects but with greater mechanical stability. For instance, arrays of gold nanodisks on glass substrates have enhanced the growth of cyanobacterium *S. elongatus* (Fig. 4c)^56^. The plasmonic substrates reflected 35% of the red light transmitted through a dilute culture back into the reactor while allowing other wavelengths to be transmitted. This approach allowed for photosynthetically useful light to be returned to the culture while permitting shorter wavelengths to be transmitted for use in photovoltaics^56^. Energy loss from ohmic resistance in plasmonic metals however, remains a significant challenge for plasmonic technologies and, together with cost, will limit the usefulness of plasmonic light management in photobioreactors going forward in all except very niche applications.

## Managing light distribution

### Culture density and path length

Photosynthetic organisms are limited in the rate at which they utilize absorbed light. When light intensity drops below a certain compensation intensity, photosynthesis is outpaced by respiration and the organism will become a net consumer of oxygen and high energy compounds to maintain metabolism through the dark period. This dark respiration for cell maintenance and biomass synthesis often results in a net decrease in biomass as carbohydrates are consumed to support these cellular processes, but not replenished through photosynthesis. The rate of dark respiration relative to growth varies between species but is typically between 20 and 30% of an organism's growth rate at the beginning of a dark period and declines with increasing dark exposure time, over the course of hours^57^. Similarly, under high irradiances, light saturation can occur. Saturation intensities vary widely but are typically around 150–400 μmol_PAR_ m^−2^ s^−1^ (refs 58, 59). When the saturation limit is reached, excess energy is dissipated as heat and fluorescence^60^, and many species will initiate photo-protection mechanisms to prevent a buildup of harmful reactive oxygen species produced by photosynthesis^41^,^60^. Because most microalgal species saturate at intensities close to 10% of peak sunlight, during much of the day they operate at low photosynthetic efficiency—absorbing but not effectively using incoming radiation. To manage high intensities, biomass density, light path length and mixing rates can be selected to optimize productivity and mitigate the effects of photoinhibition from excess light.

**Figure 5:**
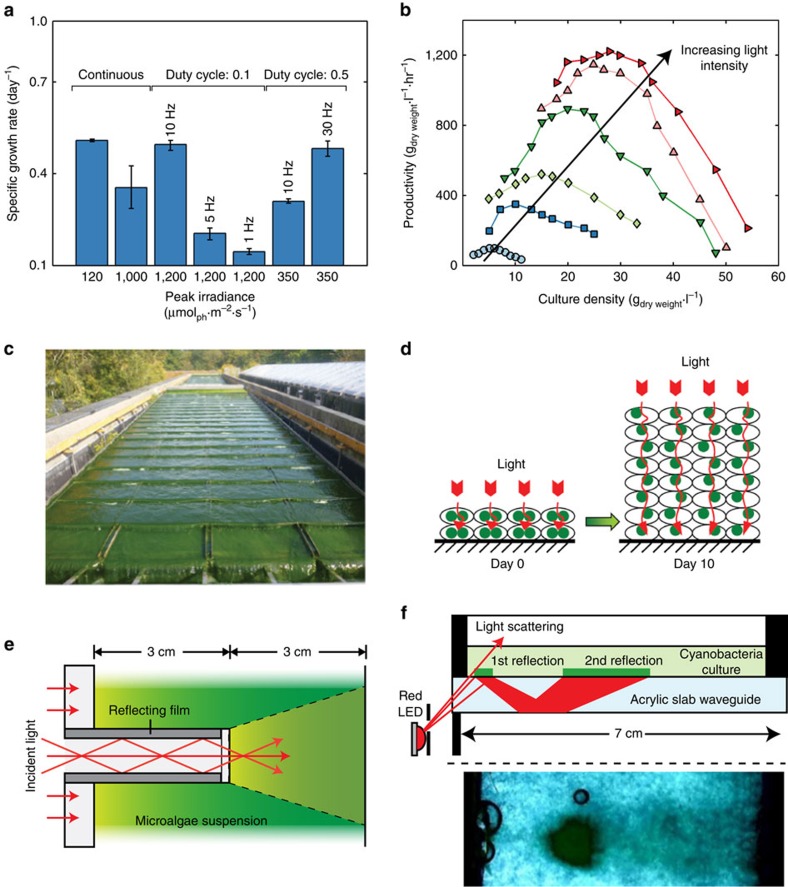
Managing light distribution. (**a**) Effects of pulsed light on the specific growth rate of *Nannochloropsis*. The duty cycles (fraction of time the light is on in a given cycle) for 1,200 μmol_ph_ m^−2^ s^−1^ and 350 μmol_ph_ m^−2^ s^−1^ pulsed light is 0.1 and 0.5 to achieve an equivalent time-averaged intensity with continuous light at 120 μmol_ph_ m^−2^ s^−1^. (**b**) Graph showing the optimal culture density under different light irradiances (circles—270 μmol_ph_ m^−2^ s^−1^, squares—740 μmol_ph_ m^−2^ s^−1^, diamonds—1,200 μmol_ph_ m^−2^ s^−1^, downwards triangle—2,000 μmol_ph_ m^−2^ s^−1^, upwards triangle—6,000 μmol_ph_ m^−2^ s^−1^, rightward triangle, 8,000 μmol_ph_ m^−2^ s^−1^). (**c**) Short-light-path cascade photobioreactor for high-density outdoor cultivation. (**d**) Schematic showing how cells in biofilms adapt to high-intensity light by reducing their concentration of light-absorbing pigments. This reduction in pigment concentration results in a more transparent biofilm which allows light to penetrate deeper allowing the biofilm to grow thicker and support more cells. (**e**) Schematic showing how light guiding elements can be used to increase the illuminated surface area to volume ratio inside of photobioreactors, reducing the irradiance seen by the culture below the saturation intensity. (**f**) Schematic of micro-scaled light delivery through waveguides to attached biofilms. Light from an LED is coupled into a slab waveguide and undergoes total internal reflection. Where cells are present on the waveguide surface, light can be scattered out or coupled directly into the photosynthetic apparatus, driving photosynthesis. The inset below shows experimental growth patterns achieved using this design. (**a**) Adapted from ref. 67, (**b**) adapted from ref. 72 with permission from Taylor&Francis Ltd, (**c**) reproduced from ref. 73 with permission from Elsevier, (**d**) reproduced from ref. 79, copyright Wang *et al*.; licensee BioMed Central 2015, (**e**) adapted from ref. 84 with permission from Elsevier, (**f**) adapted with permission from ref. 86, copyright 2014 IOP Publishing Ltd.

While no universal rules for optimal path length and density apply across all species, some general trends have emerged that result in improved performance. Under high-intensity sunlight, optimal productivity often results from a combination of path length, density and mixing rate such that the frequency of cycling between the light and dark regions is on the same timescale as the turnover rate of the photosynthetic machinery, an effect demonstrated in several species^61^,^62^,^63^,^64^,^65^. It has been proposed that the flashing light effect can mitigate photoinhibition by providing a dark period of sufficient length to allow the electron transporters involved in shuttling electrons between reaction centres time to reoxidize^41^,^58^, thereby avoiding the need to exhaust energy through non-photosynthetic quenching pathways. Efficiency generally increases with pulse frequencies of 10–100 Hz (Fig. 5a)^61^. Similarly for saturating light, pulse times on the order of 1–10 ms followed by dark periods on the order of tens of milliseconds tend to be optimal^61^,^62^,^63^,^66^. In this way, productivity approaching the productivity of continuous light at the same time-averaged intensity can be achieved (full-light integration), even when the absolute intensity of the pulsed light is beyond the saturating intensity of the organism. Because the time-averaged intensity is necessarily less than the surface irradiance, the overall photosynthetic efficiency for the culture will be greater.

Studies exploring the flashing light effect have typically relied on pulsed LED light to simulate the cycling of cells between light and dark zones in a photobioreactor and have shown full-light integration to occur for species such as *C. reinhardtii* at frequencies greater than 50 Hz (ref. 61), and 10 Hz for *Nannochloropsis salina*^67^. The conditions under which light integration occurs and its extent is expected to vary between species and only a limited number have been evaluated. The diatom, *Phaeodactylum tricornuturn*, for instance, has achieved full-light integration at the much lower frequencies of 1 Hz (ref. 68). In photobioreactors where light–dark cycling is a function of geometry, cell density, and mixing, practical cycle frequencies are limited to less than 5 Hz (refs 64, 69, 70). In these contexts, only partial light integration is expected to occur, but can nevertheless result in a meaningful improvement in productivity and efficiency provided that culture density, path length and mixing rate are selected in concert to provide the optimal light regime and hydrodynamics^64^,^67^,^71^,^72^. Figure 5b shows an example of how productivity depends, for instance, on culture density for different light intensities^72^. High-density cultures and path lengths on the order of millimetres have been recommended to achieve the necessary cycle frequencies. Outdoor, high-density (6 g L^−1^) cascade reactors cultivating *Chlorella sp*., similar to the one shown in Fig. 5c^73^, have shown considerable improvements in productivity over more dilute installations and operate with culture depths of 6mm resulting in cycle frequencies of around 0.5 Hz (ref. 74). It has also been suggested that designing the system such that the time-averaged light intensity is close to but not exceeding the saturating intensity of the cells would maximize productivity and photosynthetic efficiency^61^.

Although alternating light–dark cycles have been shown to improve productivity for several species of interest, for others it has shown only marginal improvement^75^,^76^,^77^. As yet it is unclear whether these empirical observations are characteristic of the species, or due to variations in experimental conditions including reactor configuration, mixing strategy, species-dependent maintenance energy requirements, spectral distribution and cultivation history. Second order effects such photo-acclimation and protection mechanisms can further obfuscate the optimization process^78^. For instance, cells immobilized in high-density biofilms and exposed to high light showed a reduction in chlorophyll concentration, reducing absorption in the biofilm and allowing light to penetrate deeper and dynamically changing the illumination profile (Fig. 5d)^79^. Further research and consistency in experimental techniques is required to bring clarity to the mixed empirical results available to date.

### Light dilution

An optical approach to managing high-intensity light is to dilute it over a larger surface area, thus reducing the local light intensity to productive levels. The curved surfaces of tubular photobioreactors are perhaps the simplest approach and can dilute incident light by a factor of 1.57 (refs 80, 81). Reactor orientation, as discussed previously, can also be adjusted to dilute light over a larger surface area of the reactor by changing the angle of the illuminated surface relative to the sun, such that the intensity will be proportional to the cosine of this angle. Obliquely incident light incurs additional losses related to Fresnel reflections at the interface between the environment and reactor surfaces. These losses are greatest at larger incident angles and at interfaces between materials with large refractive index contrasts, such as air (*n*=1) and glass (*n*=1.5).

Incorporation of transparent panels or waveguides into the culture volume is another light dilution approach (Fig. 5e)^82^,^83^,^84^. Biofilm photobioreactors (in which cells are cultivated as surface attached colonies rather than in suspension, (Fig. 5f) can particularly benefit from larger surface areas since more illuminated area is available for attachment and cultivation^85^,^86^. Roughened optical fibres have been used for this purpose to distribute light to biofilms attached to the fibre surfaces, and achieved light energy to hydrogen conversion efficiencies of 9.3% compared with 3.9% for cultures in suspension^87^. Similarly, stacks of optical waveguides have been used to distribute light to ultra-high-density cultures with path lengths of 2 mm and achieved an eightfold improvement in productivity over bulk cultures with a path length of 30 mm (ref. 85). Achieving uniform scattering of light from waveguides is desirable and can be achieved through patterned or random surface treatments^88^, though complete uniformity may not be necessary provided the peak intensity is below the saturation threshold.

## Managing cellular light utilization

Engineering the molecular structure of the cellular light-harvesting apparatus presents genetic-engineering opportunities for efficiency improvement^89^. Excitation energy transfer to the reaction centres happens within 100 ps with efficiencies of 80%–100% (ref. 90), however, the slower enzyme-mediate reactions involved with water oxidation, electron transport and carbon fixation, can introduce bottlenecks particularly under high light. These bottlenecks can be avoided by reducing the photon absorption rate, which can be accomplished by reducing the size and absorption cross-section of the light-harvesting complex^91^.

For sub-saturating intensities, photosynthetic efficiency can be improved by increasing the spectral sensitivity of the light-harvesting apparatus to utilize a larger portion of the spectrum. This can be accomplished by engineering the pigment composition of the light-harvesting complexes making more wavelengths available for photosynthesis^89^. Control over spectral sensitivity as well as photo-protective mechanisms (which tend to direct energy towards non-photosynthetic channels) can be achieved by synthetically accessorizing light-harvesting antennae with additional carotenes and xanthophylls^90^. This approach represents a synthetic variation of the natural photo-adaptive responses of plants and microalgae in which pigments re-organize in response to changing light conditions^92^. Making more photons available for photosynthesis is advantageous provided photoinhibition by light saturation is avoided; strategies that rely on increasing absorption must simultaneously manage the incident photon flux.

Light harvesting and energy transduction at the cellular level can also leverage recent advances in nanomaterials. For example, single-walled carbon nanotubes have been inserted and irreversibly localized within the lipid envelope of isolated plant chloroplasts to assist with electron transport^93^. The result was a threefold increase in photosynthetic activity compared with unmodified chloroplasts. Light-harvesting nanocrystals have been grown on the surface of non-photosynthetic bacteria to generate electron–hole pairs and transfer these excited carriers directly to the cell for use in acetic acid synthesis^94^. Conversion efficiencies close to 80% were achieved under low light intensity by utilizing highly absorbing nanocrystals that feed charge-carriers to the high efficiency Wood-Ljungdahl acetic acid synthesis pathway. These studies, while still at the proof-of-concept stage, point towards the emerging opportunities for nanotechnology-augmented photosynthesis.

Alternative avenues of solar conversion via photosynthesis are emerging, many of which draw inspiration from nature in an engineering-biology-materials partnership. For example, charge-carriers generated through microalgal photosynthesis have been captured and used directly to power electrical devices^9^. The efficiency of these bio-photovoltaic cells, while still relatively low, is increasing, and may approach that of other photobiological and photovoltaic technologies^9^. Another hybrid system combines silicon nanowire arrays with bacteria to generating acetic acid^95^. Reducing equivalents generated by solar energy incident on the silicon nanowires are used by surface adhered bacteria to metabolize CO_2_. Future embodiments involving multiple, genetically tailored, bacteria could then use this acetic acid as a substrate to produce a range of useful polymers, biofuels and pharmaceutical precursors^95^.

## Perspectives

Efficiently navigating the conversion of sunlight into value-added products is a key challenge for commercially viable bioproducts from microalgae, in particular for biofuels where margins are low. Incumbent global and regional energy systems are entrenched with decades and even centuries of experience, innovation, investment and infrastructure. The unprecedented success of fossil fuels puts a tremendous burden on emerging technologies in a market that is not fully motivated to seek alternatives. Fortunately, low-value biofuels can be co-produced with higher value but low-volume products^14^. For example, microalgae are a source of polyunsaturated fatty acids which are used in nutraceuticals, animal feedstocks, infant formulas and aquaculture. Carotenoids such as beta-carotene and astaxanthin are useful as food colourants and nutraceuticals. Phycobilins, which occur exclusively in microalgae, are valuable as fluorescent markers for laboratory and therapeutic purposes, and as natural food and cosmetic colourants. Biodegradable polymers can be made from polyhydroxyalkanoate, a polyester expressed by some bacteria and genetically modified macroalgae^96^. Co-production of useful high-value products with high-energy-density fuels can support the continued development of economical cultivation and processing technologies. Co-production is also an advantage photosynthesis demonstrates over other solar-fuel generation techniques. For instance photovoltaic-powered water electrolysis which has a similar theoretical energy conversion efficiency of around 13% (20% photovoltaic energy conversion efficiency, 65% electrolyser efficiency) is constrained to produce only hydrogen. Other photovoltaic-powered electrolysis such as electro-reduction of CO_2_ produces CO as main product with a solar-to-fuel conversion efficiency lower than 10% (ref. 97). Similarly, while photovoltaic cells have achieved over 40% efficiency, electricity is untenable for many applications particularly those requiring high-energy density such as transportation or long-term storage.

The ultimate practicality of each of the techniques discussed in this review is a function of implementation feasibility, cost and product value. Specifically, site selection considerations have much in common with photovoltaic light harvesting, and synergies between these two fields should be exploited, including design and modelling tools. Artificial light, in particular LEDs, are not currently practical for commodity production, but the production of high-value, low-volume compounds is feasible in the near-term and will support technology maturation. Further work to understand the combined effects of light spectrum distribution, intensity and physical/chemical environments is needed for a wider range of species, using consistent and systematic methods. To conduct these experiments, in high numbers and with the needed control, parallelization will be required beyond that of flask-based approaches applied to date^65^. Wavelength shifting materials hold a promise to increase the fraction of sunlight available for photosynthesis, though practical implementations which avoid reabsorption and scattering losses have not been forthcoming. Improved materials and incorporation of these materials into innovative reactor geometries are required to push photosynthetic efficiencies beyond the current theoretical limits. Other approaches to light spectrum management, such as plasmonic scattering, will only be viable provided that significant modification of the light spectrum can be achieved while internal losses remain low. This is a challenge in many optical systems, but plasmonics in particular.

Looking forward, we see three major opportunities for integrating the best photon management strategies in developing photobioreactor operations. First, advanced materials for customizing the spectrum of light incident on the cultures will enable production of specific bioproducts. So far, these effects have been studied in only a relatively small number of organisms and additional work is needed to understand the effects of spectral distribution on a wider range of organisms. Second, the economic potential of microalgal cultivation will be made clearer by designing cultivation schemes which are integrated with infrastructures able to provide supplies of carbon and nutrients, such as flue gas or municipal wastewater systems. This type of integration and context-specific assessment will quantify the ancillary benefits of mitigating and utilizing waste streams, adding value to the microalgal cultivation process. Further integration and development of on-site biomass processing technology such as hydrothermal liquefaction of algal biomass can provide insight into the full life-cycle efficiency of microalgal biofuel production. Third, better coordination and partnerships between pilot scale plant operators and academia will avail the latest developments to industry, provide for more consistent data collection and reporting of key performance metrics, and focus the efforts of academia on the specific constraints affecting the success of early pilots.

## Additional information

**How to cite this article:** Ooms, M. D. *et al*. Photon management for augmented photosynthesis. *Nat. Commun.* 7:12699 doi: 10.1038/ncomms12699 (2016).

## Figures and Tables

**Table 1:** Microalgae cultivation metrics.

**Quantity**	**Units**
Photosynthetic efficiency	% [*E*_product_·*E*_incident light_^−1^]
Photosynthetic quantum efficiency	mol_fixed carbon_·mol_photons_^−1^
Areal productivity	g_product_·m^−2^·per day
Volumetric productivity	g_product_·L^−1^ per day
Product yield on light	g_product_·mol_photons_^−1^

**Table 2:** Light management strategies in photobioreactors.

**Strategy**	**Effects**	**References**
Reactor location selection	Solar irradiance and trajectory changes with latitude, with the highest irradiances near the equator. Weather patterns also greatly affect the amount of sunlight available to solar energy harvesting installations.	13,15,16,17,18,19,20,21
Orientation and solar tracking	East–West vertical facing surfaces intercept more light, but cast shadows limiting the advantage for large area installations.	22,23,24,25,26,27,28,80
Light spectrum effects	Changing the spectral distribution of light can help to maximize growth by increasing the amount of photosynthetically active radiation and/or stimulate the expression of valuable metabolites.	30,32,33,35,36,37,38
Wavelength shifting	Converting light of low photosynthetic utility into light of high utility can make more energy available for photosynthesis.	45,46,47,48,49,50,51
Artificial light	Light-emitting diodes provide spectral, temporal and spatial control of light. For the production of high-value compounds, the convenience and flexibility of LED illumination may justify their associated energy and capital costs.	33,40,42,44
Plasmonic scattering	Plasmonic scattering or nearfield confinement can be used to selectively direct or confine useful wavelengths of light into the reactor.	52,53,54,55,56
Culture density and light path length	The light regime within a photobioreactor directly impacts the rate of photosynthesis and respiration. Controlling the cell density, light path, and mixing rates can result in optimal areal productivity and efficiency. In particular, short path lengths coupled with high-density cultures and mixing can cycle cells between light and dark zones, increasing efficiency of outdoor cultures when irradiances are above the saturation limit.	41,58,61,62,63,64,65,66,67,68,70,71,72,73,74,75,76,77,78,79
Light dilution	High-intensity light can be distributed over larger surface areas using light guiding elements or employing curved surfaces to dilute the light to an intensity below the saturation threshold of the microalgae.	77,80,81,82,83,84,85,86,87,88
Cellular engineering	New opportunities to improve the efficiency and utility of microalgae are presenting themselves through engineering at the cellular level. These enhancements can be purely biological, as in the genetic modification of light-harvesting antenna size, or a combination of biology with synthetic materials, as in the use of nanotubes or quantum dots to improve energy transduction.	89,90,91,93,94,95

## Figures of merit used in microalgal cultivation.

Typical metrics used to evaluate microalgae cultivation are presented in Table 1, **productivity** being the primary performance metric for cultivation systems. **Areal productivity** is the amount of biomass or product generated per unit area of facility space, or per unit area of illuminated surface. **Volumetric productivity** is the mass of product produced per unit of culture volume. **Photosynthetic yield on light** measures the ratio of the mass of product generated per mole of photons intercepted by the culture (g_product_·mol_photons_^−1^). Knowing the energy content of the products, one can calculate the overall photosynthetic efficiency *E*_product_·*E*_incident light_^−1^. For biomass, the energy content varies by type but is typically around 23 kJ·g _dry weight_^−1^ (ref. 43) or 480 kJ mol^−1^ of fixed carbon^11^. **Photosynthetic efficiency** describes the energy conversion efficiency of incident optical energy into stored chemical energy and relates the intensity of light to the productivity of a cultivation system. Maximum theoretical values for photosynthetic efficiency have been estimated for outdoor culture and typically range between 8% and 12% based on full spectrum sunlight. In literature, photosynthetic efficiency is often reported with respect to PAR and can consequently seem artificially high. Similar ambiguity is introduced when photosynthetic conversion efficiency is calculated based on artificial light sources with diverse spectra and care must be taken when making comparisons.

For consistency within the field and with other fields involved in solar energy utilization, photosynthetic energy conversion efficiency is reported herein based on total incident energy.
